# A population‐based analyses of the evolving management of cN1M0 prostate cancer in the PSMA‐PET era

**DOI:** 10.1002/bco2.70059

**Published:** 2025-07-21

**Authors:** Jennifer Ward, Kevin Armstrong, Therese Min‐Jung Kang, Jodie Tham, Yuan‐Hong Lin, Marcus Cheng, Jeremy Grummet, Nathan Lawrentschuk, Marlon Perera, Shomik Sengupta, David Chang, Michael Ng, Jonathan Bensley, Michelle Steeper, Krupa Krishnaprasad, Maggie Johnson, Nikolajs Zeps, Jeremy Millar, Wee Loon Ong

**Affiliations:** ^1^ Alfred Health Radiation Oncology, School of Translational Medicine Monash University Melbourne VIC Australia; ^2^ Department of Radiation Oncology, Olivia Newton‐John Cancer and Wellness Research Centre Austin Health Heidelberg VIC Australia; ^3^ Department of Radiation Oncology, Andrew Love Cancer Centre Barwon Health Geelong VIC Australia; ^4^ Department of Urology Alfred Health Melbourne VIC Australia; ^5^ Department of Urology Royal Melbourne Hospital VIC Australia; ^6^ Division of Cancer Surgery Peter MacCallum Cancer Centre Melbourne VIC Australia; ^7^ EJ Whitten Prostate Cancer Research Centre Epworth Healthcare Melbourne VIC Australia; ^8^ Department of Urology Austin Health Heidelberg VIC Australia; ^9^ Department of Urology Eastern Health Box Hill VIC Australia; ^10^ Eastern Health Clinical School Monash University Box Hill VIC Australia; ^11^ Department of Radiation Oncology Peter MacCallum Cancer Centre Melbourne VIC Australia; ^12^ Sir Peter MacCallum Department of Oncology University of Melbourne VIC Australia; ^13^ Genesis Cancer Care Victoria Melbourne VIC Australia; ^14^ Prostate Cancer Outcomes Registry Victoria (PCOR‐VIC), School of Public Health and Preventive Medicine Monash University Melbourne VIC Australia

**Keywords:** androgen deprivation therapy, node positive, prostate cancer, prostatectomy, PSMA‐PET, radiation therapy

## Abstract

**Objectives:**

To evaluate the patterns of management of clinical node‐positive non‐metastatic prostate cancer (cN1M0PC) at a population‐based level over time, and to identify factors associated with the different management approaches.

**Patients and methods:**

The study included men diagnosed with cN1M0PC in Prostate Cancer Outcome Registry Victoria (PCOR‐Vic) in Australia between 2008 and 2022. The primary outcome was the use of local therapy (radical prostatectomy or prostate+/− pelvic radiation therapy) within the first 12 months of diagnosis. Multivariable logistic regressions were used to evaluate factors associated with local therapy use among all men and the likelihood of having a radical prostatectomy among men who had local therapy.

**Results:**

Of the 819 men included in this study, 52% had PSMA‐PET staging, and this increased over time to 74% in 2018–2022. There were 530 (65%) who had local therapy (169 radical prostatectomy, and 361 radiation therapy), 259 (32%) had systemic therapy alone, and 30 (4%) did not have any treatment. There was an increase in the proportion of men who had local therapy over time, from 52% in 2008–2012 to 72% in 2018–2022. In multivariable analyses, increased age, higher PSA and residency in regional/remote areas were independently associated with lower likelihood of local therapy use, while PSMA‐PET staging and more recent year of diagnosis were associated with higher likelihood of local therapy use. Of the 530 men who had local therapy, increased age, higher PSA, higher ISUP grade group and higher clinical T categories were associated with a lower likelihood of having radical prostatectomy while men diagnosed in private institutions and from higher socioeconomic quintiles were more likely to have radical prostatectomy.

**Conclusion:**

This is the largest contemporaneous population‐based study on the management of cN1M0PC in the PSMA‐PET era. There is an increasing use of local therapy for cN1M0PC over time, with large variations in practice.

## 1 INTRODUCTION

The presence of regional nodal involvement represents a poor prognostic indicator for prostate cancer and is associated with higher recurrence rates, increased distant metastatic disease and lower survival rates.^1^ Historically, men with prostate cancer with nodal involvement were considered to harbour systemic disease, hence, they were generally not treated with curative intent and were most commonly managed with androgen deprivation therapy (ADT) alone. However, several large multi‐institutional retrospective studies^2^ and population‐based studies^3^, ^4^, ^5^, ^6^ have suggested that local therapy with radical prostatectomy or radiation therapy for clinical node‐positive non‐metastatic prostate cancer (cN1M0PC) improves cancer outcomes. Subgroup exploratory analyses from the STAMPEDE trial have also suggested an oncological benefit with the addition of radiation therapy to the prostate and regional node in men with cN1M0PC.^7^ There is, however, no prospective randomized data on the optimal management of cN1M0PC.^8^ Based on the limited available evidence, the 2024 NCCN guidelines and the EAU guidelines have recommended that local therapy (with surgery or radiation therapy) are both appropriate management options for men with cN1M0PC who have life expectancies of over 5 years.^9^ Hence, there is potentially large variations in the patterns of clinical practice in the management of cN1M0PC.

A concurrent advancement in prostate cancer care that may have impacted on evolving management of cN1M0PC is the advent of novel imaging such as prostate‐specific membrane antigen positron emission tomography (PSMA‐PET). PSMA‐PET imaging has been shown to have superior accuracy in the detection of nodal or distant metastases compared to conventional imaging with computed tomography (CT) or bone scan in the proPSMA trial.^10^ PSMA‐PET has allowed for earlier detection of hitherto unsuspected cN1M0PC, which would otherwise have been considered node‐negative (N0) prostate cancer using conventional imaging.^10^, ^11^ Similarly, PSMA‐PET has also allowed for earlier detection of low volume metastatic prostate cancer, which would have otherwise been considered non‐metastatic (M0) prostate cancer on conventional imaging. PSMA‐PET is now Medicare Benefits Schedule (MBS) funded as the primary staging imaging for newly diagnosed intermediate‐risk to high‐risk prostate cancer in Australia since 1 July 2022.^12^ However, PSMA‐PET was already widely available and easily accessible in Australia prior to that,^13^ through patient self‐funding, industry funded patient access scheme, public hospital availability, as well as enrolment in clinical trials such as proPSMA trial.^14^ It is unclear how, and if, the management of cN1M0PC may have changed along with the widespread availability of PSMA‐PET scans in Australia.

The aim of this study is to evaluate the evolving patterns of the management of N1M0PC at a population‐based level over time, and to identify factors associated with the different management approaches.

## 2 METHODS

### 2.1 Study population

The study comprises men registered in the Prostate Cancer Outcomes Registry Victoria (PCOR‐Vic) who were diagnosed with cN1M0PC, based on conventional imaging and/or PSMA‐PET imaging^13^ between 2008 and 2022. Details of recruitment into PCOR‐Vic have been previously published.^15^ In brief, PCOR‐Vic was established in 2008 to investigate variations in the presentation, pattern of care and outcomes of men with prostate cancer in Victoria.^16^ Men with newly diagnosed prostate cancer were notified to PCOR‐Vic, with an opt out consent process to optimize the recruitment rate. As of 2022, the population coverage for PCOR‐Vic was >70%. The data in PCOR‐Vic was collected by trained data collectors, through men's medical record review as well as patient contact at 12 months after diagnosis (for men who did not receive any treatment), or 12 months after treatment (for men who received any active treatment).

### 2.2 Primary outcomes and covariables

The primary outcome of interest in the current study is the temporal trend in the primary treatment (within the first 12 months of cancer diagnosis) for cN1M0PC over the study period. The year of diagnosis was grouped into 5‐year groups, i.e. 2008–2012, 2013–2017 and 2018–2022.

The management was broadly categorized as radical prostatectomy, radiation therapy (to prostate+/−pelvic nodes) with or without androgen deprivation therapy, systemic therapy alone (androgen deprivation therapy, and/or chemotherapy) and observation alone. *Local therapy* was defined as either radical prostatectomy or radiation therapy. All surgical treatments involved a radical prostatectomy; however, the PCOR‐Vic did not capture details on whether pelvic lymph node dissection was performed in men who had radical prostatectomy. Similarly, details on radiation therapy techniques (e.g. 3‐dimensional conformal radiation therapy, intensity modulated radiation therapy, or volumetric modulated arc therapy), as well as details relating to the radiation therapy target volume (e.g. prostate alone or prostate and pelvic nodes), were not captured in the PCOR‐Vic.

Covariables included in the analyses were age at diagnosis, PSA level at diagnosis, ISUP grade group, clinical T categories, socioeconomic status, remoteness of residency and the diagnosing institutions. Socioeconomic status and remoteness, and residency were both derived from the men's residential postcodes. Socioeconomic status was generated using the Australian Bureau of Statistics (ABS) Socio‐Economic Indexes for Area (SEIFA) Index of Relative Socio‐economic Advantage and Disadvantage (IRSAD) and subdivided into quintiles based on the Australian data. The measure of remoteness of residence was classified based on the Modified Monash Model (MMM), into metropolitan and regional/remote areas. The diagnosing institutions were categorized as public and private institutions.

### 2.3 Statistical analyses

Categorical variables were summarized using frequency and percentage, while continuous variables were summarized using mean and standard deviation (or median and interquartile range, as appropriate). Cochran‐Armitage test for trend was used to evaluate the changing trend in local therapy use (and separately for radical prostatectomy and radiation therapy) over time. Multivariable logistic regressions were used to evaluate the likelihood of having local therapy among all men, and the likelihood of having radical prostatectomy among those who had local therapy. A two‐sided P‐value <0.05 was considered to indicate statistical significance. All statistical analyses were performed using Stata/MP18 (StataCorp College Station, TX, USA).

## 3 RESULTS

### 3.1 Study population

**FIGURE 1:**
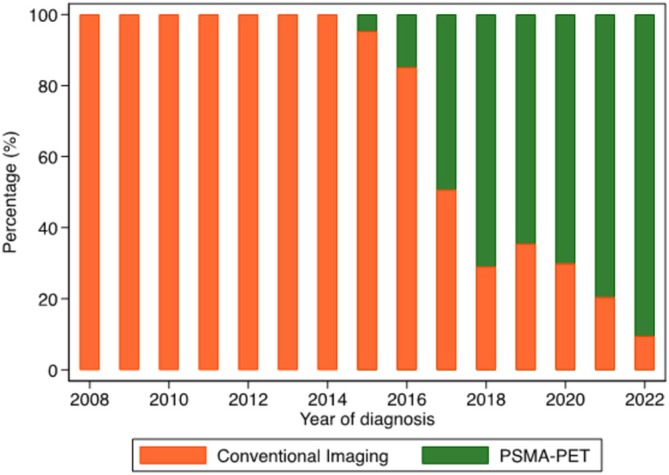
Proportion of men with clinical node‐positive non‐metastatic prostate cancer (cN1M0PC) who had staging with conventional imaging vs PSMA‐PET imaging from 2008 to 2022.

There were 819 men diagnosed with cN1M0PC between 2008 and 2022 and registered in the PCOR‐Vic who were included in this study (Figure S1). Baseline characteristics are listed in Table 1. The mean and median age at diagnosis was 71.3 years (SD = 8.4) and 71.3 years (IQR = 65.1–77.0). Approximately 40% of men had PSA of >20 ng/l at diagnosis, and clinical T3/4 disease, and more than 50% had ISUP grade group 5 disease. Half of the men were diagnosed in public and private institutions, respectively. Approximately two‐third of the men lived in metropolitan areas. Half of the men in the study had PSMA‐PET staging, and this increased significantly over time (P‐trend <0.001) and by 2022, 90% of men in the study had PSMA‐PET staging (Figure 1).

**TABLE 1:** Baseline characteristics of study population (n = 819).

**Age (years)**	
Mean (SD)	71.3 (8.4)
Median (IQR)	71.3 (65.1–77.0)
< 60 years	70 (9%)
60–69 year	281 (34%)
70–79 years	340 (42%)
≥80 years	128 (16%)
**Serum PSA at diagnosis (ng/mL)**	
Median (IQR)	16.1 (8.5–38)
< 10 ng/mL	245 (30%)
10–20 ng/mL	191 (23%)
> 20 ng/mL	338 (41%)
Missing	45 (5%)
**ISUP Grade Group**	
Group 1	12 (1%)
Group 2	63 (8%)
Group 3	113 (14%)
Group 4	141 (17%)
Group 5	441 (54%)
Missing	49 (6%)
**Clinical T categories**	
T1	89 (11%)
T2	158 (19%)
T3/4	330 (40%)
Missing	242 (30%)
**PSMA PET staging**	
No	389 (47%)
Yes	430 (53%)
**Diagnosing institution**	
Public	398 (49%)
Private	419 (51%)
Missing	2 (0.2%)
**Socioeconomic status**	
Quintile 1 (lowest)	122 (15%)
Quintile 2	103 (27%)
Quintile 3	159 (19%)
Quintile 4	180 (22%)
Quintile 5 (highest)	254 (31%)
Missing	1 (0.1%)
**Remoteness of residence**	
Metropolitan areas	534 (65%)
Regional/remote area	285 (35%)
**Year of diagnosis**	
2008–2012	69 (8%)
2013–2017	236 (29%)
2018–2022	514 (63%)

### 3.2 Local therapy

**FIGURE 2:**
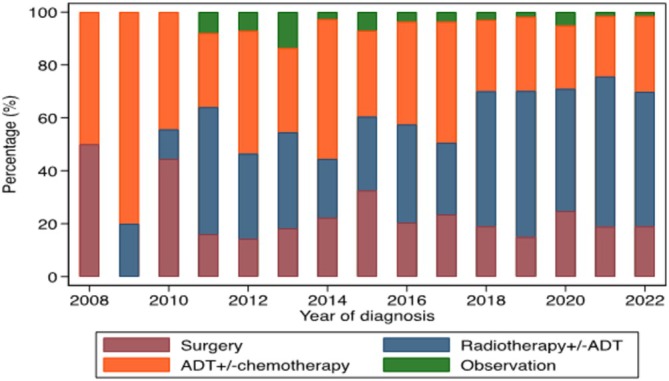
Management approaches for men with clinical node‐positive non‐metastatic prostate cancer (cN1M0PC) from 2008 to 2022.

Of the 819 men included in this study, 530 (65%) had local therapy with either radical prostatectomy (n = 169) or radiation therapy (n = 361), at a median of 1.6 months (IQR = 1.0–2.6 months) after diagnosis. Of the men who had radiation therapy, 344 (95%) had ADT, started at a median of 4.7 months (IQR = 3.5–5.9 months) prior to radiation therapy. There were 259 (32%) men treated with systemic therapy alone (androgen deprivation therapy +/− chemotherapy), and 30 (4%) did not have any treatment within the first 12 months of diagnosis. Overall, there was an increase in the proportion of men who had local therapy over time (P‐trend < 0.001), with a statistically significant increase in the trend of radiation therapy (P‐trend <0.001), but no statistically significant change in the trend for radical prostatectomy (P‐trend = 0.5) (Figure 2).

**FIGURE 3:**
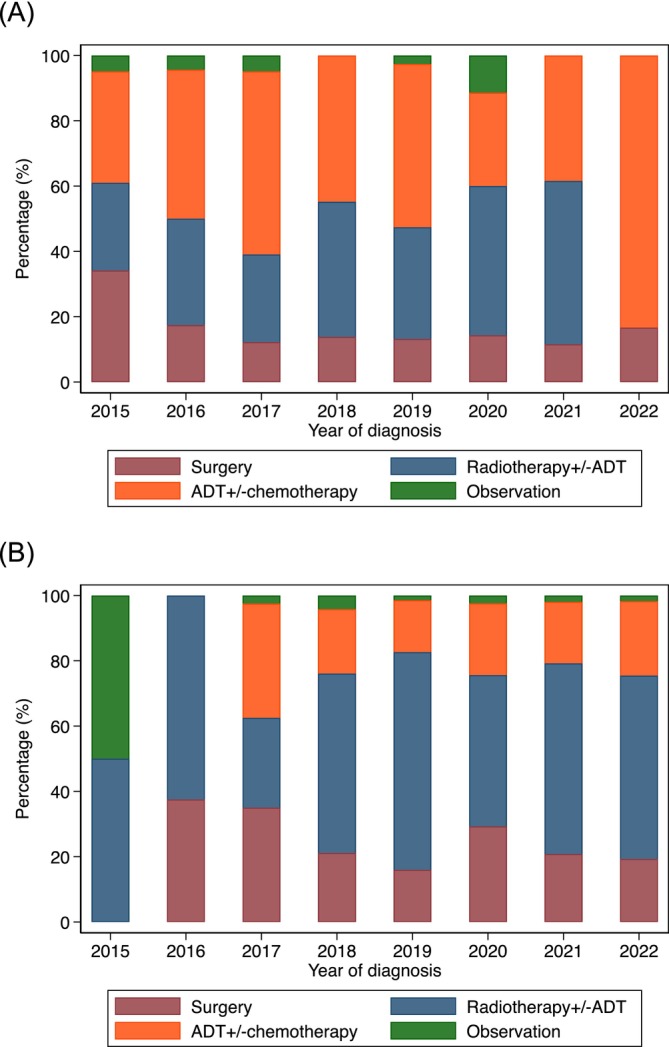
Management approaches for clinical node‐positive non‐metastatic prostate cancer (cN1M0PC) for men who had conventional imaging alone (A) and PSMA‐PET imaging (B) from 2015 to 2022. *only included data from 2015 when PSMA‐PET staging is available.

Men who had local therapy were younger (mean age 69.7 years) compared to those who did not have local therapy (mean age 74.3 years) (Table 2). Men with lower PSA (<10 ng/ml) were more likely to have local therapy (82%), compared to 70% of men with PSA 10‐20 ng/ml and 51% of men with PSA > 20 ng/ml. Higher proportion of men who had PSMA‐PET staging (77%) had local therapy, compared to those who did not have PSMA‐PET staging (51%) (Figure 3). A higher proportion of men diagnosed in private institutions (70%) had local therapy compared to those diagnosed in public institutions (60%). Across different socioeconomic quintiles, 60–70% of men had local therapy. A higher proportion of men who live in metropolitan areas (67%) had local therapy compared to those who live in regional/remote areas (60%).

**TABLE 2:** Proportion of men who had local therapy and the likelihood of having local therapy for clinical node‐positive non‐metastatic prostate cancer (cN1M0PC) in multivariable analyses (n = 819).

	No local therapy (n = 289, 35%)	Local therapy (n = 530, 65%)	OR (95%CI)	P‐value
**Age at diagnosis**			0.94 (0.92–0.97)	<0.001
Mean (SD)	74.3 (8.9)	69.7 (7.7)		
Median (IQR)	74.9 (68.2–81.2)	70.3 (64.5–74.9)		
**Serum PSA at diagnosis**				
Median (IQR)	29.0 (13.7–69.0)	12.6 (7.7–26.8)		
< 10 ng/mL	45 (18%)	200 (82%)	Reference	
10–20 ng/mL	57 (30%)	134 (70%)	0.48 (0.27–0.86)	0.01
> 20 ng/mL	165 (49%)	173 (51%)	0.33 (0.19–0.56)	<0.001
**ISUP Grade Group**				
Group1–3	54 (29%)	134 (71%)	Reference	
Group 4	45 (32%)	96 (68%)	0.83 (0.43–1.57)	0.6
Group 5	155 (35%)	286 (65%)	0.86 (0.52–1.43)	0.6
**Clinical T categories**				
T1/2	70 (28%)	177 (72%)	Reference	
T3/4	104 (32%)	226 (68%)	1.22 (0.79–1.89)	0.4
**PSMA PET**				
No	189 (49%)	200 (51%)	Reference	
Yes	100 (23%)	330 (77%)	1.80 (1.07–3.01)	0.03
**Diagnosing institution**				
Public	160 (40%)	238 (60%)	Reference	
Private	127 (30%)	292 (70%)	1.33 (0.86–2.06)	0.2
**Socioeconomic status**				
Quintile 1 (lowest)	45 (37%)	77 (63%)	Reference	
Quintile 2	41 (40%)	62 (60%)	1.38 (0.60–3.19)	0.4
Quintile 3	53 (33%)	106 (67%)	0.77 (0.37–1.63)	0.5
Quintile 4	71 (39%)	109 (61%)	0.55 (0.26–1.16)	0.1
Quintile 5 (highest)	79 (31%)	175 (69%)	0.75 (0.36–1.59)	0.5
**Remoteness of residence**				
Metropolitan areas	176 (33%)	358 (67%)	Reference	
Regional/remote areas	113 (40%)	172 (60%)	0.56 (0.33–0.94)	0.03
**Year of diagnosis**				
2008–2012	33 (48%)	36 (52%)	Reference	
2013–2017	110 (47%)	126 (53%)	1.27 (0.63–2.56)	0.3
2018–2022	146 (28%)	530 (72%)	2.37 (1.10–5.10)	0.03

OR = odds ratio.

In multivariable analyses, age at diagnosis, serum PSA at diagnosis, PSMA‐PET staged, areas of residence and the year of diagnosis were independently associated with the likelihood of having local therapy (Table 2). Older men were less likely to have local therapy (OR = 0.94; 95%CI = 0.92–0.97; P < 0.001). Compared to men with PSA < 10 ng/ml, those with PSA 10‐20 ng/ml (OR = 0.48; 95%CI = 0.27–0.86; P = 0.01) and >20 ng/ml (OR = 0.33; 95%CI = 0.19–0.56; P < 0.001) were less likely to have local therapy. Men who had PSMA‐PET staging were 1.8 times (95%CI = 1.07–3.01; P = 0.03) more likely to have local therapy compared to men who did not have PSMA‐PET staging. Men living in regional/remote areas were less likely to have local therapy compared to men living in metropolitan areas (OR = 0.56; 95%CI = 0.33–0.94; P = 0.03). Men diagnosed in 2018–2022 were 2.4 times (95%CI = 1.10–5.10; P = 0.03) more likely to have local therapy compared to men diagnosed in 2008–2012.

### 3.3 Type of local therapy

Of the 530 men who had local therapy, 169 (32%) had radical prostatectomy and 361 (68%) underwent radiation therapy as primary treatment (Table 3). Of the 361 men who radiation therapy, 68 (19%) had 50–60 Gy, 195 (54%) had 70‐78Gy and 98 (27%) had missing information on radiation therapy dose. Of the 169 men who had radical prostatectomy, 48 (28%) had post‐operative radiation therapy. Men who had a radical prostatectomy were younger (mean age 65.5 years), compared to those who had radiation therapy (mean age 71.6 years). Men with higher PSA levels were more likely to have radiation therapy – 82% men with PSA > 20 ng/ml had radiation therapy vs 18% who had radical prostatectomy. Men with higher ISUP grade groups were more likely to have radiation therapy – 76% and 72% of men with ISUP grade groups 4 and 5, respectively, had radiation therapy. There were 81% men with cT3/4 prostate cancer who had radiation therapy, compared to 19% who had radical prostatectomy. A higher proportion of men diagnosed in private institutions had radical prostatectomy (43%) compared to those diagnosed in public institutions (17%). Men from the highest socioeconomic quintiles were more likely to have radical prostatectomy (45%), compared to those from the lowest socioeconomic quintiles (25%).

**TABLE 3:** Proportion of men who had radical prostatectomy vs radiation therapy, and likelihood of having radical prostatectomy for clinical node positive non‐metastatic prostate cancer (cN1M0PC) in multivariable analyses (n = 530).

	Radiation therapy (n = 361, 68%)	Radical prostatectomy (n = 169, 32%)	OR (95%CI)	P‐value
**Age at diagnosis**			0.86 (0.83–0.90)	<0.001
Mean (SD)	71.6 (7.4)	65.5 (6.6)		
Median (IQR)				
**Serum PSA at diagnosis**				
Median (IQR)	14.8 (8.3–31.0)	9.2 (6.2–17.0)		
< 10 ng/mL	111 (55%)	89 (45%)	Reference	
10–20 ng/mL	92 (69%)	42 (31%)	0.57 (0.29–1.11)	0.1
> 20 ng/mL	142 (82%)	31 (18%)	0.20 (0.10–0.41)	<0.001
**ISUP Grade Group**				
Group 1–3	70 (52%)	64 (48%)	Reference	
Group 4	73 (76%)	23 (24%)	0.49 (0.21–1.14)	0.1
Group 5	206 (72%)	80 (28%)	0.47 (0.25–0.89)	0.02
**Clinical T categories**				
T1/2	99 (56%)	78 (44%)	Reference	
T3/4	183 (81%)	43 (19%)	0.43 (0.24–0.77)	0.004
**PSMA PET**				
No	129 (65%)	70 (35%)	Reference	
Yes	232 (70%)	99 (30%)	0.99 (0.48–2.06)	0.9
**Diagnosing institutions**				
Public	197 (83%)	41 (17%)	Reference	
Private	164 (56%)	128 (43%)	4.68 (2.52–8.66)	<0.001
**Socioeconomic status**				
Quintile 1 (lowest)	58 (75%)	19 (25%)	Reference	
Quintile 2	50 (81%)	12 (19%)	1.42 (0.42–4.77)	0.6
Quintile 3	81 (76%)	25 (24%)	1.35 (0.45–4.03)	0.6
Quintile 4	76 (70%)	33 (30%)	2.10 (0.71–6.20)	0.2
Quintile 5 (highest)	96 (55%)	79 (45%)	3.38 (1.18–9.68)	0.02
**Remoteness of residence**				
Metropolitan areas	235 (65%)	123 (34%)	Reference	
Regional/remote area	126 (73%)	46 (27%)	1.89 (0.88–4.05)	0.1
**Year of diagnosis**				
2008–2012	23 (64%)	13 (36%)	Reference	
2013–2017	70 (56%)	56 (44%)	1.49 (0.48–4.67)	0.5
2018–2022	268 (73%)	100 (27%)	0.76 (0.23–2.56)	0.7

In multivariable analyses, age, serum PSA at diagnosis, ISUP grade group, clinical T categories, diagnosing institutions and socioeconomic status were independently associated with a likelihood of having radical prostatectomy. Compared to men with PSA < 10 ng/ml, those with PSA > 20 ng/ml were less likely to have radical prostatectomy (OR = 0.20; 95%CI = 0.10–0.41; P < 0.001). Compared to men with ISUP grade group 1–3, those with ISUP grade group 5 were less likely to have radical prostatectomy (OR = 0.47; 95%CI = 0.25–0.89; P = 0.02). Men with higher clinical T categories were less likely to have radical prostatectomy (OR = 0.43; 95% CI = 0.24–0.77; P = 0.004). Men diagnosed in private institutions were 4.7 times (95%CI = 2.52–8.66) more likely to have radical prostatectomy. Men from the highest socioeconomic quintiles were 3.4 times (95%CI = 1.18–9.67) more likely to have radical prostatectomy compared to men from the lowest socioeconomic quintiles.

## 4 DISCUSSION

This study evaluates the evolving patterns in the management of cN1M0PC over a 15‐year period from 2008 to 2022 at a population‐based level in Victoria, Australia. To the best of our knowledge, this stands as the largest and most contemporaneous cohort of men with cN1M0PC in Australia. There are several important findings in this study.

Firstly, we observed that two‐thirds of men with cN1M0PC in Victoria had local therapy, with approximately two‐thirds treated with radiation therapy and another one‐third treated with radical prostatectomy. The overall use of local therapy increased over time from approximately 52% in 2008–2012 to 72% in 2018–2022. The most marked increase was observed in the use of radiation therapy. This is likely based on exploratory subset analyses in patients with cN1M0PC in the STAMPEDE trial, whereby 82% of men received radiation therapy to the prostate and pelvic nodes and had improved oncological outcomes compared to those who did not receive radiation therapy – 2‐year failure‐free survival 81% vs 53%.^7^ Several previous US population‐based studies have evaluated the pattern of management of cN1M0PC, albeit over a much earlier study period. In a study using data from the Surveillance, Epidemiology and End Results (SEER) database between 1995 and 2005, Rusthoven et al reported 43% (340/796) men with cN1M0PC had local therapy with radiation therapy.^3^ Separately, in a slightly more modern series between 2006 and 2011 using data from the National Cancer Database (NCDB), Moon et al identified 8464 men with cN1M0PC, of which ADT alone was the most common management approach (46%), while 3379 (40%) had local therapy – 722 (9%) with radical prostatectomy and 2657 (31%) with radiation therapy.^17^ The use of local therapy in our cohort is slightly higher at approximately 50% in 2008–2012 even when we looked at similar and overlapping period with the NCDB study cohort.

The observation of increasing local therapy use for cN1M0PC over time in our study needs to be interpreted in the settings of advancement in staging imaging for prostate cancer,^18^ with almost three‐quarters of men in the study had PSMA‐PET staging in the 2018–2022 period. Australia is among the world leaders in the introduction of PSMA‐PET imaging for prostate cancer. The proPSMA trial has shown that the use of PSMA‐PET staging has resulted in a change in management in approximately one‐third of men with prostate cancer.^10^ However, it remains unknown and speculative as to whether these PSMA‐PET staged cN1M0PC simply represents earlier detection of cN1M0PC with same biology disease had they been staged with conventional imaging at a later stage, and hence had a higher likelihood of ‘cure’ with earlier local therapy, or that they potentially reflect a more unique and potentially more favourable PSMA‐sensitive biology process compared to non‐PSMA expressing prostate cancer.^19^, ^20^ Regardless, the resultant stage migration for cN1M0PC due to advancement of PSMA‐PET staging may, in time, artificially translate into improved outcomes for PSMA‐PET staged cN1M0PC, a phenomenon known as the Will Rogers effect.^21^, ^22^ Separately, given that the access to PSMA‐PET scans in the earlier study period was likely limited to large tertiary centres, the association between PSMA‐PET staging and local therapy use may also reflect patients' access to urological or radiation oncology expertise in these tertiary centres, who may be among the earlier adopters of local therapy for cN1M0PC.

Secondly, we identified several factors to be associated with the use of local therapy for cN1M0PC. After adjusting for tumour characteristics (PSA, clinical T categories and ISUP grade group), we observed that older men and those who lived in regional/remote areas were less likely to have local therapy. Older men were likely to have a more limited life expectancy and may be more appropriately managed with a watchful waiting approach (i.e. observation) or ADT alone. The association of age and local therapy use for cN1M0PC has been previously reported in a study using the NCDB database.^17^ The reduced likelihood of men in regional areas having local therapy may reflect reduced access to radical prostatectomy and/or radiation therapy, which may have influenced the decisions made regarding local therapy for cN1M0PC. In a study from the US NCDB database of >200,00 with prostate cancer, it has been reported that approximately one‐in‐two men from urban areas who live within 5 miles of radiation therapy facilities had radiation therapy, compared to only approximately one‐in‐four men from rural area who live >75 miles from radiation therapy facilities.^23^ Another possibility is that men living in regional areas may potentially have more advanced disease, which were unaccounted for in our multivariable analyses (e.g. more extensive nodal involvement as opposed to solitary nodal involvement), which may have influenced the decision on local therapy recommendations.

Of the men who had local therapy, we observed differences in radical prostatectomy or radiation therapy use depending on age, socioeconomic group and diagnosing institutions, after adjusting for tumour characteristics. Older men were less likely to have radical prostatectomy, which may reflect general fitness or suitability for surgery. Men from the highest socioeconomic quintiles and those who were diagnosed in private institutions were more likely to have radical prostatectomy. While there is exploratory analyses from prospective trial suggesting the benefits of radiation therapy for cN1M0PC,^7^ there is no randomized trial comparing surgery vs radiation therapy for cN1M0PC. The observed differences in the use of various local therapies is likely multifactorial, and may also reflect men’ preferences. In an earlier New South Wales study of >4000 men with prostate cancer in the 45 and Up Study cohort, it has been reported that men from higher socioeconomic groups and those with private health insurance were more likely to have radical prostatectomy over radiation therapy.^24^

There are several limitations in this study. PCOR‐Vic database is a population‐based database and lacks the granularity for us to comment on the appropriateness of local therapy in individual case. There is a lack of data on comorbidities, performance status and life expectancy estimates, baseline urinary symptoms, as well as other details in terms of contraindications for radical prostatectomy or radiation therapy – all of which may influence the decision on local therapy and the type of recommended local therapy. There is also a lack of data on the treatment details, e.g. whether lymph nodal dissection was performed, as well as the radiation therapy treatment fields used. Men with cN1M0PC are also a heterogenous group, which could range from those having solitary pelvic nodal involvement to extensive pelvic nodal disease, which is not captured in PCOR‐Vic database, and this may influence the recommendation on the appropriateness of local therapy. We also cannot discount the possibility of misclassification of cN1M0PC in the PCOR‐Vic database, given that nodal involvement could potentially be classified as cN1 (nodal involvement in true pelvis i.e. below the bifurcation of common iliac vessels) versus cM1a (nodal involvement beyond the bifurcation of the common iliac vessel), and the classification of cN1 vs cM1a disease can sometimes be equivocal. Beyond the pattern of practice itself, of greater interest is whether the uptake of local therapy (and the use of different local therapy) actually translates into improved oncological outcomes. Unfortunately, this data is not captured and available in PCOR‐Vic, and relevant outcomes such as biochemical failure or distant metastases are not available in any other population‐based dataset in Australia.

Moving forward, we anticipate that the management of cN1M0PC will continue to evolve. While there is no currently recruiting surgical trials for cN1M0PC, there are several ongoing radiation oncology trials that may provide us with more evidence‐based management for cN1M0PC in the future.^8^ These include: The UK PEARLS trial (CRUK/19/016; ISRCTN36344989), a phase 2/3 trial which includes individuals with cN1M0PC randomized to radiation therapy to the prostate and pelvic nodal +/− extended field including para‐aortic nodal chain, along with ADT,^25^ and the NRG‐Oncology HIGH‐FIVE trial (NCT05946213) which includes individuals with cN1M0PC randomized to conventional fractionated or moderate hypofractionated radiation therapy vs 5‐fraction stereotactic radiation therapy to the prostate and pelvic nodes. The Trans‐Tasman Radiation Oncology Group (TROG) is working closely with international cooperative trial groups to hopefully bring some of these trials into Australia, allowing patients access to these, potentially practice‐defining, clinical trials. In the meantime, there are several Australian single‐institutional phase 2 trials in the space of cN1M0PC that is currently recruiting, e.g. the SNIPER trial (ACTRN12625000259448).

## 5 CONCLUSION

In summary, this study represents the largest population‐based cohort of men with cN1M0PC in the PSMA‐PET era in the literature, offering insight into the large variations and the evolving management practices. It reveals an increase in the use of local therapy for cN1M0PC over time, but with large variations in practice, depending on socioeconomic status, areas of residence and diagnosing institutions. It is important to recognize that there is currently a lack of high‐level robust evidence on the optimal management of cN1M0PC, which may partly explain the large variations in practice observed at a population‐based level. Nonetheless, this study has provided us with useful data to continue to monitor and benchmark our practice as PSMA‐PET imaging is becoming the standard of care primary staging modality for prostate cancer.

## AUTHOR CONTRIBUTIONS

**Wee Loon Ong:** Data curation; study concept and design; acquisition of data; analysis and interpretation of data; drafting of manuscript; critical revision of manuscript for important intellectual content; supervision; statistical analysis. **Jennifer Ward:** Study concept and design; drafting of manuscript; critical revision of manuscript for important intellectual content. **Therese Min‐Jung Kang:** Study concept and design; drafting of manuscript; critical revision of manuscript for important intellectual content. **Jeremy Millar:** Study concept and design; acquisition of data; critical revision of manuscript for important intellectual content. **Jonathan Bensley:** Acquisition of data; statistical analysis. **Kevin Armstrong:** Drafting of manuscript; critical revision of manuscript for important intellectual content. **Michelle Steeper:** Acquisition of data; critical revision of manuscript for important intellectual content. **Maggie Johnson:** Acquisition of data; critical revision of manuscript for important intellectual content. **Krupa Krishnaprasad:** Acquisition of data; critical revision of manuscript for important intellectual content.

## CONFLICT OF INTEREST STATEMENT

The author declare that they have no known competing financial interests or personal interest that could have appeared to influence the work reported in this article.

## Supporting information


**Figure S1.** Patient flow diagram of men included in the study
