# Supplementary material for: A population‐based analyses of the evolving management of cN1M0 prostate cancer in the PSMA‐PET era
Source: BJUI Compass. 2025 Jul 21;6(7):e70059. doi: 10.1002/bco2.70059 (PMC12277652; doi:10.1002/bco2.70059)
Supplement: Supplementary file 1 — Figure S1. Patient flow diagram of men included in the study [file BCO2-6-e70059-s001.docx]

**Supplementary Figure – 1 | Patient flow diagram of men included in the study**

40,849 men diagnosed with prostate cancer between January 2008 and December 2022 in PCOR-Vic

Excluded:

33,269 with node negative (N0) prostate cancer

3,044 with metastatic disease (M1)

Excluded

38 unclear management

819 with documented management details

857 men who had node positive non-metastatic prostate cancer (N1M0)
